# Near-Complete Genome Sequences of Streptomyces sp. Strains AC1-42T and AC1-42W, Isolated from Bat Guano from Cabalyorisa Cave, Mabini, Pangasinan, Philippines

**DOI:** 10.1128/MRA.00904-18

**Published:** 2018-08-23

**Authors:** Marian P. De Leon, A-young Park, Andrew D. Montecillo, Maria Auxilia T. Siringan, Albert Remus R. Rosana, Song-Gun Kim

**Affiliations:** a Microbial Culture Collection, Museum of Natural History, University of the Philippines Los Baños, Laguna, Philippines; b Biological Resource Center, Korean Collection for Type Cultures, Korea Research Institute of Bioscience and Biotechnology, Jeongeup, Republic of Korea; c Departament de Produccio Vegetal I Ciencia Forestal, Universitat de Lleida, Lleida, Spain; d Microbiological Research and Services Laboratory, Natural Sciences Research Institute, University of the Philippines Diliman, Quezon City, Philippines; e Department of Chemistry, University of Alberta, Edmonton, Alberta, Canada; University of Maryland School of Medicine

## Abstract

Streptomyces sp. strains AC1-42T and AC1-42W, isolated from bat guano from Cabalyorisa Cave, Mabini, Pangasinan, Philippines, are active against Bacillus subtilis subsp. subtilis KCTC 3135^T^. The near-complete genome sequences reported here represent a possible source of ribosomally synthesized, posttranslationally modified peptides, such as lantipeptides, bacteriocins, linaridin, and a lasso peptide.

## Abstract (precis)

Streptomyces sp. strains AC1-42T and AC1-42W, isolated from bat guano from Cabalyorisa Cave, Mabini, Pangasinan, Philippines, are active against Bacillus subtilis subsp. subtilis KCTC 3135^T^.

## ANNOUNCEMENT

The genus Streptomyces, the largest in the phylum *Actinobacteria*, represents the bacterial gold standard for secondary metabolite mining and a major source of medically important antibiotics (1). Streptomyces sp. strains AC1-42T and AC1-42W were isolated from bat guano from Cabalyorisa Cave, Mabini, Pangasinan, Philippines, and were preserved at the Microbial Culture Collection, Museum of Natural History (MCC-MNH 1948 and 1949, respectively). AC1-42T cultured on tryptic soy agar produced a translucent colony, while AC1-42W produced a white colony, and both strains showed antagonistic activity against Bacillus subtilis subsp. subtilis KCTC 3135^T^. Here, we report the near-complete genomes of Streptomyces sp. strains AC1-42T and AC1-42W for genome-assisted discovery of novel bioactive secondary metabolites with potential antibacterial, antitumor, or antiviral properties.

Genomic DNA was extracted from cells grown on tryptic soy broth (30°C, 72 h) using a NucleoSpin microbial DNA kit (Macherey-Nagel, Germany) and sequenced at Macrogen, Inc. (Seoul, Republic of Korea). High-molecular-weight DNA (14 μg and 12 μg for AC1-42T and AC1-42W, respectively) was used to construct libraries that were size selected to generate 20-kb SMRTbell templates, annealed using the PacBio DNA polymerase binding kit P6, and sequenced using the PacBio version 4.0 sequencing kit using 8 single-molecule real-time (SMRT) cells (C4 chemistry) on the PacBio RS II platform (Pacific Biosciences, USA). *De novo* assembly of PacBio reads for AC1-42T (179,039 reads; 1,507,227,395 bases; *N*_50_ = 11,738 bp) and AC1-42W (149,211 reads; 1,428,149,357 bases; *N*_50_ = 13,289 bp) was performed using the Hierarchical Genome Assembly Process 3 (HGAP3) and Falcon pipelines. The final genome size of AC1-42T is 7.27 Mbp in five contigs (*N*_50_ = 2,461,044) with 71.5% G+C content and a sequencing depth of 118×. AC1-42W has a 7.65-Mbp genome in four contigs (*N*_50_ = 3,945,785), 71.6% G+C content, and a depth of 135×. Annotation of AC1-42T predicted 6,206 coding sequences, 16 rRNAs, and 60 tRNAs, while AC1-42W revealed 6,420 coding sequences, 17 rRNAs, and 72 tRNAs using the Prokaryotic Genome Annotation Pipeline (PGAP) (2). Whole-genome comparison was performed using the Microbial Genome Atlas (MiGA) (3) utilizing the NCBI prokaryotic databases. Species identity was established by calculating the average nucleotide identity (ANI) and digital DNA-DNA hybridization (dDDH) using the ANI calculator (4) and the Genome-to-Genome Distance Calculator (GGDC) version 2.1 (5), respectively. Secondary metabolites were mined using antiSMASH version 4 (6), lasso peptides were mined using Rapid ORF Description and Evaluation Online (RODEO) (7), and bacteriocins were mined using BAGEL4 (8).

The genomes closest to AC1-42T and AC1-42W were those of Streptomyces sp. strains SirexAA-E (ANI, 84%) and PBH53 (ANI, 80%), respectively. The genomes of AC1-42T and AC1-42W were predicted to have 59 and 65 biosynthetic gene clusters (BGCs), respectively. These include ribosomally synthesized, posttranslationally modified peptides, such as linaridin, lasso peptide, lantipeptides, and thiopeptide. The genomes also encoded BGCs for terpenes, nonribosomal peptides, butyrolactones, and nonribosomal peptide-polyketide hybrids. The genomes contain BGCs supporting the biosynthesis of other metabolites, including the antitumor nataxazole (9), the osmolyte ectoine (10), and the isoprenoid hopene (11). Two antibiotics, tetronomycin (12) and a thiazolyl peptide, are unique to strain AC1-42W. Future validation of these bioactive compounds may be pursued using activity-guided purification or heterologous expression.

### Data availability.

The whole-genome sequences of Streptomyces sp. strains AC1-42T and AC1-42W have been deposited in DDBJ/ENA/GenBank under the accession numbers QKWX00000000 and QKWY00000000, respectively.
